# Prenatal Exposure to Nitrogen Oxides and its Association with Birth Weight in a Cohort of Mexican Newborns from Morelos, Mexico

**DOI:** 10.29024/aogh.914

**Published:** 2018-07-27

**Authors:** Jessica Mendoza-Ramirez, Albino Barraza-Villarreal, Leticia Hernandez-Cadena, Octavio Hinojosa de la Garza, José Luis Texcalac Sangrador, Luisa Elvira Torres-Sanchez, Marlene Cortez-Lugo, Consuelo Escamilla-Nuñez, Luz Helena Sanin-Aguirre, Isabelle Romieu

**Affiliations:** 1 Instituto Nacional de Salud Pública, Av. Universidad # 655, Col. Santa María Ahuacatitlán, C.P. 62100 Cuernavaca, Morelos, MX; 2 Facultad de Enfermería y Nutriología, Universidad Autónoma de Chihuahua, Circuito Universitario Campus II, C.P. 31240 Chihuahua, Chih, MX; 3 Centro de Investigación en Materiales Avanzados S.C., Complejo Industrial Chihuahua, Avenida Miguel de Cervantes 120, C.P. 31109 Chihuahua, Chih, MX; 4 Facultad de Ingeniería, Universidad Autónoma de Chihuahua, Circuito Universitario Campus II, C.P. 31240 Chihuahua, Chih, MX

## Abstract

**Background:**

The Child-Mother binomial is potentially susceptible to the toxic effects of pollutants because some chemicals interfere with placental transfer of nutrients, thus affecting fetal development, and create an increased the risk of low birth weight, prematurity and intrauterine growth restriction.

**Objective:**

To evaluate the impact of prenatal exposure to nitrogen oxides (NO_x_) on birth weight in a cohort of Mexican newborns.

**Methodology:**

We included 745 mother-child pair participants of the POSGRAD cohort study. Information on socio-demographic characteristics, obstetric history, health history and environmental exposure during pregnancy were readily available and the newborns’ anthropometric measurements were obtained at delivery. Prenatal NO_x_ exposure assessment was evaluated using a Land-Use Regression predictive models considering local monitoring from 60 sites on the State of Morelos. The association between prenatal exposure to NO_x_ and birth weight was estimated using a multivariate linear regression models.

**Results:**

The average birth weight was 3217 ± 439 g and the mean of NO_x_ concentration was 21 ppb (Interquartile range, IQR = 6.95 ppb). After adjusting for maternal age and other confounders, a significant birthweight reduction was observed for each IQR of NO_x_ increase (ß = –39.61 g, 95% CI: –77.00; –2.21; p = 0.04).

**Conclusions:**

Our results provides evidence that prenatal NO_x_ exposure has a negative effect on birth weight, which may influence the growth and future development of the newborn.

## Introduction

An accelerated increase in health effect related to air pollution has been observed over recent years in developed as well as developing countries [1,2,3,4]. Various studies have reported that air pollution exposure is associated with a deterioration in the health of the exposed population, particularly in terms of respiratory [5], cardiovascular [6,7] and reproductive effects [8,9,10,11,12,13]. The mother-child pair is potentially susceptible to the toxic effects of pollutants since certain chemicals can interfere with the placental transfer of nutrients and affect the fetal development [14,15], thereby increasing the risk of low birth weight, premature birth, intrauterine growth restriction, and/or a combination thereof.

Birth weight is an indicator of the duration of gestation and fetal growth rate [16], and is a determinant of childhood morbidity and mortality, as well as, morbidity in adulthood [17], A newborn’s weight depends of several factors, including those related to the infant, as well as, maternal, hereditary, and environmental conditions, such as air pollution exposure, whose association with birth weight has been previously studied, however the results have been contradictory and have not been conclusive [18,19].

The primary source of air pollutants and NO_x_ emissions is vehicle exhaust, which contributes to 64% of total emissions [20]. In Mexico, the relationship between air pollutants and respiratory health has been well documented [3,21,22], however, actually, none studied have evaluated the role of prenatal NO_x_ exposure on reproductive effects considering a birth cohort study.

Therefore, we realized the present study to evaluate the impact of prenatal exposure to nitric oxide (NO), nitrogen dioxide (NO_2_) and nitric oxides (NO_x_) on birth weight in a cohort of newborns from Morelos, Mexico.

## Material and Methods

### Study Design and Population

This analysis is based on the POSGRAD (**P**renatal **O**mega-3 **S**upplementation on child **GR**owth **A**nd **D**evelopment) cohort study; a large double blind randomized controlled trial (RCT) of prenatal DHA supplementation. Pregnant women (n = 1094) were randomized to receive a daily supplement of 400 mg of DHA or placebo from 18–22 weeks of pregnancy until delivery. A detailed description of the design and methods has been published elsewhere [23]. Briefly, eligible women were 18–35 years of age, at 18–22 weeks of gestation, planned to deliver at the Mexican Institute of Social Security General Hospital in Morelos, Mexico and planned to live in the area for 2 years after delivery. A total of 1094 women were randomly assigned to the clinical trial and for the present report we included 745 binomials, which had the complete information.

The study protocol was approved by the National Institute of Public Health Biosafety, Investigation, and Ethics Committees and for the Emory University Institutional Review Board. Written informed consent was obtained from participating mothers after they received a detailed explanation of the study.

### Collection of Information

*Prenatal Period*: At the first prenatal check-up, trained staff members administered a structured questionnaire to collect information about sociodemographic characteristics, obstetric history, and maternal health, including: consumption of drugs, active smoking, pre-gestational weight and size, consumption of maternal vitamins, and information about the pregnancy evolution. During the prenatal period, a visit to the participants’ homes was performed and through a questionnaire and direct home observation, we obtained information regarding the characteristics of the household, indoor (passive smoking, fuel used for cooking and/or heating the home, etc.), and outdoor exposure to pollutants (whether they lived in industrial or agricultural zones, distances from roads with heavy vehicular traffic, etc.).

*At birth*: Using an *ad hoc* form, personnel staff obtained information about the newborn characteristics, including: condition at birth (live or stillbirth), gender, type of birth, complications at birth, presence of congenital abnormalities, and gestational age. Birth weight was obtained according to the technique proposed by Lohman [24], using a TANITA “mommy and baby” scale with a precision of ±20 grams.

### Exposure Assessment

For each participant, we estimated the individual NO_x_ exposure during pregnancy at home, using a standardized area specific land-use regression (LUR) models. The LUR models were based on measurements of NO_2_, NO and NO_x_ for a continuous period of 15 days in 60 different sites throughout the State of Morelos. Ambient levels of air pollutants were measured with Ogawa and 3M passive samplers. The samplers were positioned outdoors, near the participants’ homes (e.g. roofs, light-poles) making sure they were not being blocked by any object that could obstruct the flow of air (e.g. trees, buildings). The samplers were cleaned before use, and transported in sealed amber-color containers before and after the measurement. After 2 weeks of continuous monitoring, the samples were collected and again placed in resealable bags in amber-colored containers and transported at 5°C at laboratory of the Mexico National Institute of Public Health where, inside a glove box, the pads were extracted and stored in refrigeration until their analysis. As part of quality control, 10% were blanks and duplicates. NO_x_ concentrations were determined at the Harvard School of Public Health, using spectrophotometry [25].

LUR models were developed for each pollutant in each study area to predict air pollution levels at the residences of the cohort participants using information about: traffic variables (type of roads and highways avenues), weather data (precipitation, wind speed, and temperature), geography (elevation and coordinates), population density and land-use variables (household, industrial, commercial, and services) [26] obtained from Geographic Information Systems (ArcGIS 9.1) [27]. Air pollution measurements were performed in 2009, but the relevant exposure window (second or third trimester of pregnancy) for development of birth outcomes extends further back in time. We therefore extrapolated air-pollution concentrations predicted by the LUR models around 2005–2007. After finding the best predictive LUR models, these were validated using goodness and fit tests (evaluation of residuals, influence points, etc.). Additionally, we evaluate the correlation between the values predicted by the LUR models and those obtained from the monitoring fixed stations.

### Statistical Analysis

To evaluate the quality and consistency of the data, an exploratory and univariate analysis was performed, and measures of central tendency and frequency were estimated for each of the study variables. A bivariate and multiple linear regression models were run to evaluate the impact of prenatal exposure of air pollutants (NO, NO_2_ and NO_x_) on the weight of newborns. As potential confounders, we evaluated: parity, maternal passive or active smoking during pregnancy, pre-gestational maternal anthropometric characteristics, intervention group among others. All of them remained in the final model except those who did not change the crude association between NO_x_ with birthweight. All of the statistical analyses were performed using Stata 13 statistical analysis software for Windows [28].

## Results

Table 1 shows selected maternal and infant characteristics. Maternal mean age was 26.3 ± 4.7 years, 39.8% of women had 7 to 12 years of education, which is consistent with the national average. 63.5% were multiparous and most of them used vitamin supplements (96.78%) and were non-smokers (98.26%). Since our exclusion criteria, the mean weight at birth was 3,217 ± 440 g and gestational age mean was 39.1 ± 1.7 weeks. In terms of age, parity, occupation, and education, the women included were not significantly different than those were excluded due to the lack of information needed to estimate exposure.

**Table 1:** Characteristics of the study population included in the study, Morelos, Mexico.

Characteristics	n = 745	%	Percentiles	

			P25	P75

**Mother’s Age (years)**				

Mean ± SD	26.3 ± 4.7		22.6	29.9
Education (years)				
≤6	13	1.74		
7 to 12	297	39.81		
13 to 15	148	19.84		
>15	288	38.61		
Parity > 1	474	63.54		
Height (cm)				
Mean ± SD	155.3 ± 5.7		152	159
Pre-gestational Weight				
Mean ± SD	61 ± 10.9		53.5	67.3
Body Mass Index*				
Mean ± SD	25.2 ± 4		22.3	27.7
Vitamin Supplements				
Yes	722	96.78		
Smoking**				
Non-smoker	733	98.26		
Passive	304	40.75		
Active	13	1.74		
Treatment Group***				
Supplement	365	48.93		
Placebo	381	51.07		
**Newborns**				

Birth Weight (g)				
Mean ± SD	3216.9 ± 439		2970	3500
Sex (%)				
Male	399	53.49		
Female	346	46.51		
Gestational age (weeks)				
Mean ± SD	39.1 ± 1.7		38.1	40.1

*: Pre-gestational. **: During pregnancy. ***: Omega-3 fatty acid supplements during pregnancy.

Table 2 shows the pollutant concentrations estimated and weather variables for the study period. The median of NO, NO_2_ and NO_x_ were 2.01 ppb, 16.5 ppb and 21.04 ppb, respectively, and the correlation between the measurements and the duplicates was .9985 (p = 0.0015) for NO_x_ and 0.9211 (p = 0.0789) for NO_2_. The values of the blanks used during the monitoring campaign ranged from 0 to 0.2. The average wind speed was 2.51 m/sec and the temperature was 20.3°C during the study period. The correlation between predicted and observed values for each of the pollutants was statistically significant (p < 0.01), and were 0.91 for NO, 0.79 for NO_2_, and 0.94 for NO_x_ (data not shown).

**Table 2:** Estimated* Nitrogen Oxides Concentrations during study period, Morelos, Mexico.

Pollutants	n	Mean ± SD	p25	Median	p75

NO ppb	745	2.7 ± 2.04	1.2	2.01	4.1
NO_2_ ppb	735	19.9 ± 23.8	10.7	16.5	20.8
NO_x_ ppb	731	19.6 ± 5.6	16.2	21.04	23.5
Wind speed m/sec	745	2.51 ± .10	2.4	2.5	2.6
Temperature °C	745	20.3 ± 2.64	19	20.5	22.4

* By predictive land use regression models.

Table 3 presents the results from the evaluation of the association between prenatal exposure to NO_x_ and birth weight. After adjusting for mother’s age, height and passive smoking, gestational age, and gender of child, a significant birth weight decrease was observed by each increment in the interquartile range of NO_x_ (ß = –39.61, 95% CI –77.0; –2.21 g; p = 0.04) and NO (ß = –42.5, 95% CI –82.73; –2.18 g; p = 0.04). No association between prenatal NO_2_ and birth weight was observed.

**Table 3:** Association (coefficient per interquartile range increase) between prenatal exposure to nitrogen oxides and birth weight of newborns from Morelos, Mexico.

Pollutants	Birth weight* (g)		

	β**	CI 95%	p-value

NO (n = 745)	–42.46	–82.73; –2.18	0.04
NO_2_ (n = 734)	8.06	–4.07; 20.20	0.19
NO_x_ (n = 730)	–39.61	–77.00; –2.21	0.04

*: Models adjusted for mother’s age and height, gestational age, sex, and passive smoking. **: Calculated coefficient for the interquartile range: NO = 3.29ppb, NO_2_ = 10.16 ppb and NO_x_ = 6.95 ppb.

## Discussion

The results from the present study suggest that prenatal exposure to NO_2_ and NO_x_, as estimated by land-use regression model, significantly decreases the birth weight of newborns residing in Morelos, Mexico. To our knowledge, this is the first prospective study performed in Mexico that analyzes the effects of prenatal exposure to nitrogen oxides (NO, NO_2_ and NO_x_) on birth weight using this methodology to evaluate the exposure to air pollutants. This approach provides stronger results given that most of previous studies evaluated NO_x_ exposure according to data from fixed monitoring stations.

Previous studies have reported an association between prenatal NO_x_ exposure per trimester or throughout the pregnancy and adverse effects on birth, including birth weight and/or low fetal weight and some of those studies estimated exposure using land-use regression and particulate matter variants, while others have used other types of dispersion models and local monitoring systems [29,30,31,32,33,34,35,36].

As part of INMA, a multicentric cohort study, Aguilera *et al*. found a decrease in fetal weight of 74.7 g per increase in interquartile range of NO_2_ (IQR = 12 μg/m^3^) adjusting by NO_x_ exposure of each trimester. That association was observed when the models had been adjusted for the three trimesters of pregnancy and for women who spent over 2 hours/day in non-residential outdoor areas. Nonetheless, when analyzing this data individually, no association was found between exposure during the entire pregnancy nor per trimester [35]. In addition, within the same cohort (INMA) but in Catalonia, Spain, researchers evaluated the effect of NO_2_ exposure on fetal weight during different weeks using ultrasound measurements from a sample of 562 women. When limiting the analysis to women who spent over 2 hours/day in non-residential outdoor areas (n = 255), a statistically significant weight decrease of –5.5 g was found in week 32 of gestation, and a similarly significant decrease (4.78 g) was found between weeks 20 and 32, for each increase in the IQR (13.23 μg/m^3^) [34]; however, this was not the case for the entire sample. Meanwhile, a study in California, United States, to evaluate the effect of NO, NO_2_, and NO_x_ on the risk of low birth weight at term births, using a LUR exposure model and with and without seasonal adjustment [36]. Researchers found a statistically significant increase of 5% and 7% in the risk of low birth weight for NO and NO_x_, respectively. Nevertheless, the data was obtained from an electronic database of birth certificates.

Although the mechanism through which prenatal exposure to NO_x_ affects birth weight is unclear, the association between exposure to these pollutants and decreased birth weight can be explained by some of the proposed mechanisms that involve placental circulation. NO_x_ may affect birth weight because NO_x_ can promote blood coagulation and viscosity [30], pulmonary and placental inflammation, and endothelial and vascular changes in the placenta, which can decrease uteroplacental blood flow and inhibit the oxygen and nutrients transfer [37]. As oxidants, these compounds increase the lipid peroxidation in both the maternal and fetal tissue and also stimulate the formation of methemoglobin, which would likely lead to hypoxia and hypoxemia [32]. In addition, the resultant systemic inflammation can eventually trigger sub-optimal placentation and increase the mother’s susceptibility to infections [38].

**Figure 1:**
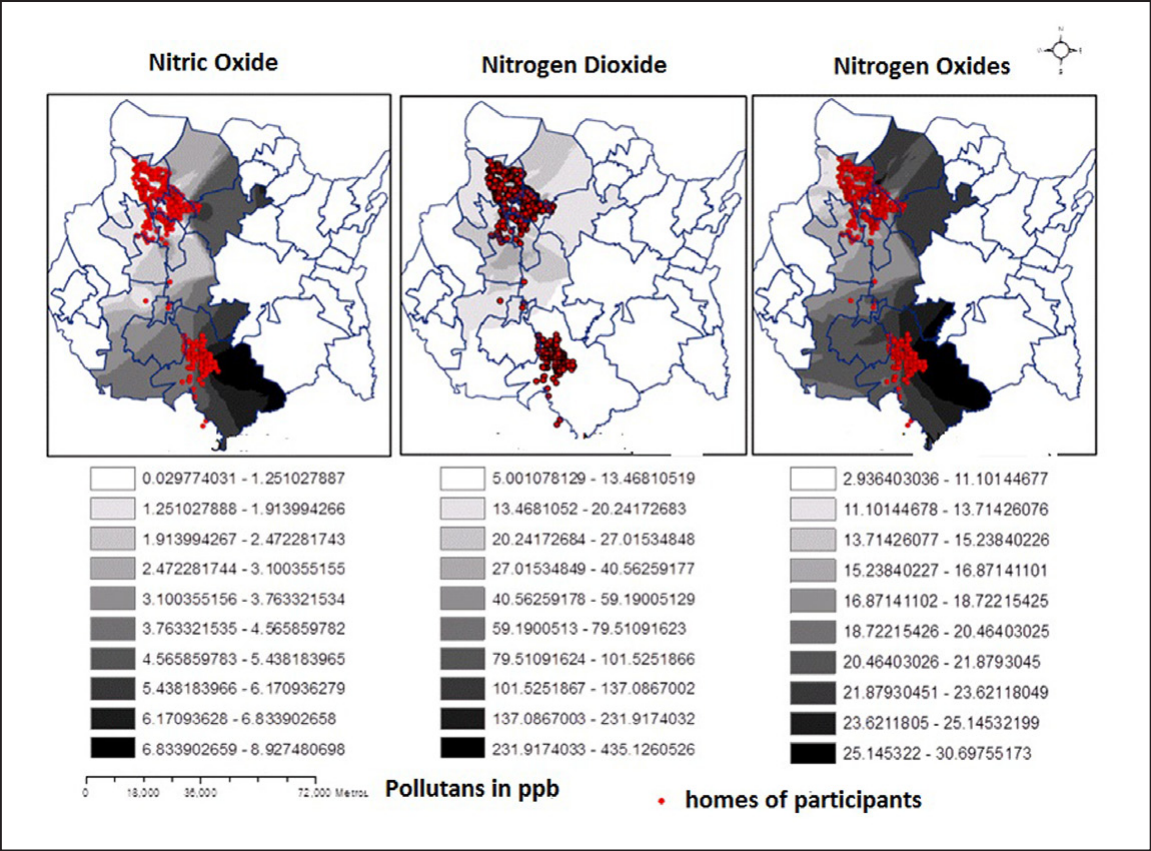
Spatial distribution of estimated nitrogen oxides concentrations in the study zone.

This study, however, has some limitations that should be considered when interpreting the results. First, the number of sites monitored and the duration of the measurements NO_x_ limits the possible variations that could occur in the concentrations of the pollutants over the study period, assuming that the air concentrations had the same behavior throughout during the entire time of pregnancy. However, the use of a LUR model to evaluate exposure strengthened the findings and made it possible to detect small-scale variations, thereby limiting classification errors in the assignment of exposure (Figure 1). It is also important to note that the correlation between the concentrations estimated by the models for the entire study period and the values obtained from the monitoring sites during the same time ranged from 0.79 to 0.95, which is statistically significant, as this information implies a correlative relationship. Furthermore, the generation of the different LUR models included weather variables for the entire monitoring period, information about variables that do not change over time, and individual characteristics of the participants.

Second, we did not obtain detailed information about the indoor and outdoor activities of each participants. Instead, we presumed that exposure was primarily associated with the amount of time spent in outdoors. However, for both cases, the measurement error was non-differential and the observed association was underestimated.

Finally, the possibility that the results were a consequence of poor control of confounders is unlikely because the design of the study excluded women with high-risk pregnancies and/or pre-existing illness and the models were adjusted for variables that were considered to have both a possible effect on birth weight and a relationship to NO_x_ exposure.

In summary, our results provide valuable information about the adverse health effect of prenatal exposure to NO_x_, especially on birth weight. These results could have significant public health implications given the role of birth weight as a determinant of childhood mortality, and more recently as a determinant of chronic illnesses during adulthood. However, it is important to continue with the investigations that allow for the identification of critical windows of exposure and that strengthen the evidence related to these associations.
